# In the eye of a quiet storm: A critical incident study on the quarantine experience during the coronavirus pandemic

**DOI:** 10.1371/journal.pone.0247121

**Published:** 2021-02-17

**Authors:** Ilaria Durosini, Stefano Triberti, Lucrezia Savioni, Gabriella Pravettoni

**Affiliations:** 1 Applied Research Division for Cognitive and Psychological Science, IEO, European Institute of Oncology IRCCS, Milan, Italy; 2 Department of Oncology and Hemato-Oncology, University of Milan, Milan, Italy; Universitat d’Alacante, SPAIN

## Abstract

**Objective:**

In 2020, the COVID-19 appeared in Italy with an exponential transmission capacity and serious consequences for the whole population. To counter the spread of the virus, the Italian government has adopted an extensive lockdown, forcing citizens to stay at home and avoid social contact. The COVID-19 quarantine represents a unique phenomenon in the recent centuries, and its long-term consequences on people’s lives and mental health are still to be understood. This study aimed to explore significant experiences of people who did not contract the virus, yet experienced the quarantine as a potentially stressful condition.

**Methods:**

Italians who did not contract the COVID-19 were invited to participate in semi-structured interviews employing the Critical Incident Technique. Interviews were designed to capture the significant experiences related to the lockdown period in Italy. Participants were asked to describe the most significant (1) negative and (2) positive critical events that they personally experienced during the ongoing quarantine. Such events were meant to provide information on their experience of the quarantine as a whole. The audio-taped interviews were transcribed verbatim and analyzed following Critical Incident Technique’s indications.

**Results:**

Twenty two participants described a total of 43 critical events, including 22 negative episodes and 21 positive events experienced during the COVID-19 quarantine. Three categories emerged from the negative episodes and four categories emerged from the positive events described by the participants. Relevant themes both positive and negative concerned mostly relationships (with partners, family, and friends), and the alteration of everyday activities, Also a specific “sensation of emergency” that the participants felt during the pandemic emerged, as an emotionally-charged response to quarantine-related external stimuli.

**Conclusions:**

To our knowledge this is the first in-depth qualitative study investigating the significant negative and positive events that people experienced during the COVID-19 quarantine. Future research could employ analogous event recollection methods but focus on other populations (e.g., fragile subjects or on other national contests), in order to extend the information on the quarantine experience and its possible long-lasting effects.

## Introduction

In early 2020, a new respiratory syndrome (COVID-19, or “Coronavirus”) appeared on the Italian territory [1, 2]. The ease of contagious through human-to-human transmission made it the sixth public health emergency of international interest as stated by the World Health Organization [3–6].

Due to the COVID-19 pandemic, from 11st March 2020 the Italian government has adopted an extensive lockdown, characterized by the closure of commercial activities, schools, universities, and by the cancellation of all the public and sports events. The Italian government also imposed a quarantine to the citizens who had to stay at home and to avoid social contacts [7, 8]. This extreme policy aimed to reduce and control the spread of the virus [9–11] which had grown exponentially within a few weeks.

However, the change in daily routine, the individual and societal financial losses related to work interruption, as well as the limitations to social contact could result in serious emotional and psychological distress [12, 13]. A recent review by Brooks and colleagues [9] has identified five stressors that could negatively influence individuals’ mental health during the quarantine: 1) duration of the quarantine; 2) fear of infection; 3) frustration and boredom; 4) inadequate supplies; 5) inadequate information.

The quarantine-related risk for psychopathology and lowering of quality of life deserves in-depth research to grasp what challenges individuals are actually facing, and also what psychological resources they are employing to manage negative consequences.

At present, it is difficult to understand how people responded to the lockdown situation, and to prefigure the social, behavioral, and emotional challenges that mental health services will have to manage in the immediate or long-term aftermath. It is easy to affirm that the lockdown and quarantine are affecting quality of life in terms of alterations caused in main areas of everyday life: **relationships** are affected because individuals are separated from their distant loved ones, or on the contrary they are obliged to spend way more time in relatives’ company when they were not used to; **work** is transforming at best (as smart working, or performed on the workplace with safety measures), or put at risk in the worst cases, leading to unemployment and serious financial crisis. Education, health management, hobbies and sports, leisure and entertainment, as activities that could hold a fundamental place in many individuals’ lives, are severely limited or completely blocked. Moreover, people could be more or less frightened about the current health emergency, living their own houses as a safe place or a prison, or even both at times, depending on personal tendencies and contingencies. What are locked down persons more unhappy about? Did the pandemic and the lockdown hit them as a traumatic experience, or as a weird, boring period where one just has to be patient? Was the lockdown even a *positive* experience for some? If yes, how?

In order to prefigure psychological support in the long-term aftermath of the COVID-19 aftermath, it is useful to collect rich data on personal stories about individuals’ life and management of the quarantine experience. It is necessary to grasp significant features of experience of people who did not contract the virus, yet experienced the quarantine in full, with significant effects on their daily routine. Such knowledge will be helpful to (1) identify which life areas (e.g., work, social relationships, hobbies and activities) have been affected by the isolation and how, so to orient future psychological support and mental health policies, and (2) understand which positive resources people have been able to employ in order to manage stressful factors, so to inform a societal comprehension of the lockdown phenomenon from the viewpoint of citizens. Moreover, lockdown and quarantine already demonstrated to influence many fields, ranging from market behavior (e.g., the increase of eCommerce utilization) [14, 15] to work and productivity (e.g., the forced digitalization of many job practices) [16, 17]. It would be useful to collect information on citizens’ behavior during the quarantine period, also to enlighten their plans, objectives and representations regarding the future.

For this reason, we adopted a qualitative research method to collect narrated events instead of abstract opinions about the quarantine, focusing on experiential and emotional accounts. We explored the experience of Italians who lived the quarantine, adopting the Critical Incident Technique (CIT) [18, 19]. It is a well-established qualitative research tool used in many studies in services evaluation [20–23], patient perspective in the healthcare context [24, 25] or meaningful life experiences [26, 27] but, to our knowledge, has still not applied to the context of the lockdown. In this study, we adapted this technique to focus on relevant positive and negative *episodes* representing individuals’ experience during the COVID-19 pandemic.

## Methods

### Recruitment and participants

During the COVID-19 outbreak, people residing in Italy, not affected by Coronavirus, were invited to describe significant events during the ongoing quarantine. Data collection was performed in April 2020, in the midst of the COVID-19 Italian pandemic.

Participants were recruited through a purposive sample and need to (a) be adults (age 20–40 years old), (b) be Italian citizens, (c) be in lockdown in Italy during the COVID-19 pandemic, and (c) not infected by COVID-19. Potential participants received the information sheet about the study and 22 adults agreed to participate (response rate: 81%). People who expressed interest in taking part in the study were contacted by a member of the research team to arrange an online interview. Prior to initiating data collection, participants were invited to give their written and verbal consent to the interview being audio-recorded and to isolate themselves from cohabitants to facilitate the disclosure of personal positive and negative events. Online interviews began after receiving participants’ written informed consent and a recorded declaration of verbal consent.

All the participants were Italian who did not contract the virus and spoke Italian as their first language. 68% of them were female with an age ranging between 21 and 37 years old (*M*_*age*_ = 29.59; *SD*_*age*_ = 3.85). The majority of the participants were well-educated (81%) and worked at home during the lockdown (64%). At the time of the interview, about half of the participants lived with their parents (41%), while the 23% of them with their partner (Table 1). All the participants were in quarantine from a minimum of 20 to a maximum of 60 days at the time of the interview (*M* = 40.36; *SD* = 11.29).

**Table 1:** Participants sociodemographic characteristics.

	*n*	%
*Gender*		
Female	15	68%
Male	7	32%
*Education*		
Primary school	0	0%
Secondary school	0	0%
High school	4	18%
Master degree	12	54%
Post-degree	6	27%
*People he/she lives with*		
Parents	9	41%
Partner	5	23%
Spouse (with or without kids)	4	18%
Alone	2	9%
Roommate(s)	2	9%

### Data collection

The Critical Incident Technique (CIT) [18] method has been used by the research team to explore the most significant events that happened to people who were experiencing the COVID-19 quarantine in Italy (see S1 Appendix for interview guide). Participants were asked to provide basic background demographic information and to describe the most significant (1) negative and (2) positive critical events that they personally experienced during the ongoing quarantine and in which they were directly involved. The open questions aimed to elicit participants’ own narratives about significant events experienced during the quarantine. Subsequently, respondents were helped to progressively move their focus from general happenings or circumstances to specific and detailed episodes.

Three researchers (ID-female, ST-male, LS-female) trained in qualitative interviewing conducted interviews via Skype and each interview was audio-recorded and lasted between 20–40 minutes. The anonymity was protected by the use of alphanumeric codes. Online interviews have been used to be able to collect data during the quarantine to facilitate recollection of recent events precisely during the period of interest. Regarding interviewers’ bias, the researchers adopted an explorative and open approach and they had no interest in the results or expectations to confirm. Researchers were in quarantine themselves when conducting the interviews, but they made sure not to influence participants to report episodes similar to their own (e.g., open and non-directive questions were used; the usage of examples to elicit episode recollection was avoided).

### Data analysis

The audio-taped interviews were transcribed verbatim and analyzed independently by two researchers (ID, ST). Following Flanagan’s indications [18], the critical incidents described by participants were considered the main unit of analysis [18, 28, 29] with the aim to “*increase the usefulness of the data while sacrificing as little as possible of their comprehensiveness*, *specificity*, *and validity*” ([18], p. 344). The following stages were followed:

1. The transcriptions were read and re-read to obtain a sense of the whole data corpus. and organised in cells in order to allow easier comparison between the participants;
2. Inductive content analysis was used to analyse the practices related to critical incidents;
3. The authors interpreted the data and constituted categories to group significant positive and negative events based on the agreement between the parties;
4. General categories and therefore more specific subcategories have been created;
5. Several categories were identified and these were then collapsed independently by the three researchers into key themes related to positive and negative critical events occurred during the quarantine.

According to Gremler [30], categories and themes emerged from data were compared, revised, and consensualized by three researchers through repeated meetings. This interactive process allows to reduce the influence of each researcher’s frames of reference and subjectivity in coding data. In this study, each critical event could be included in only one category [31]. The design and findings of this qualitative study are reported following the consolidated criteria for reporting qualitative research (COREQ; S2 Appendix) [32].

### Ethical approval

This study was approved by the University of Milan Research Ethics Committee (n. 46/20).

The permission and agreement consent was obtained from all participants before starting data collection. Prior to the interviews, participants received a copy of the information sheet containing the description of the study and the informed consent. Written and verbal consent was obtained from all participants. Confidentiality of information and privacy of participants’ interviews were respected. The anonymity of participants was protected by the use of alphanumeric codes. The participants were informed that the information collected in this study was used for this research.

## Results

Twenty two participants described a total of 43 critical events, including 22 significant negative episodes and 21 significant positive events experienced during the COVID-19 quarantine.

The categories and subthemes emerged from the data were described below (Table 2).

**Table 2:** Categories and subthemes (italicized) emerged from negative and positive events.

NEGATIVE EVENTS		POSITIVE EVENTS	
*Categories and subthemes*	*n*	*Categories and subthemes*	*n*
1. Relationships during the quarantine		1. Quarantine as an opportunity to take care of personal relationships	
*Putting relationships to the test*	*3*	*Use technologies to stay in touch with distant loved ones*	*6*
*Preoccupation for others*	*3*	*Improvement of relationships inside home*	*6*
2. Quarantine as an obstacle to movement and personal freedom		2. The working career will continue despite the pandemic	
*Limitation to movement regarding one specific event or situation during the quarantine*	*3*	*Confirmation of the employment contract*	*2*
*Alterations to important events*	*6*	*Discovery of new working activities during the quarantine*	*1*
*Impossibility to carry out daily activities during the quarantine*	*3*	3. A new value for everyday activities	
3. A sensation of emergency during the quarantine		*Modifying or discovering activities*	*4*
*The signs of COVID-19 emergency are easily identifiable in the life context*	*3*	*A sense of freedom*	*1*
*The “tilt” or chaos of systems and services due to the pandemic*	*1*	4. Quarantine as an opportunity to take a break from the normal routine	
		*Take your time*!	*1*

### Negative events

Each participant included in this study described a significant negative episode related to their quarantine outbreak. A total of 22 critical negative incidents were collected and coded. Three categories and seven sub-categories emerged from the negative episodes described by participants.

#### 1. Quarantine as an obstacle to movement and personal freedom

Out of the 22 negative events reported by participants, twelve concerned the quarantine as an obstacle to movement and personal freedom (54.5%). This category ranged from not being able to reach a specific important event or to help loved ones in need (*n* = 3), to the distress related to alterations in habits and daily activities (*n* = 3). More, six additional episodes regarded specific events that were attended by the participants, but significantly altered by the quarantine situation and safety measures.

*Limitation to movement regarding one specific event or situation during the quarantine*. During the quarantine, one participant (female, 31 years old, ID#7) remembered the most significant negative episode related to the limitation of movement due to the lockdown. She was not able to participate in her grandmother’s funeral due to the restrictions to personal movement. This led the participant to perceive the lockdown “like a prison” which prevented her to be close to relatives in a time of need: *“The police said it was not a sufficient motive to get an authorization (to move)*. *In the end there has been a small ceremony at my parents’ house*, *and I was able to attend it just by video-call (*…*) When they said I could not go it was like the whole world was falling apart*. *(*…*) At first I thought*, *I just grab a couple clothes and go*, *but then… in that moment*, *I really felt the lockdown like a prison*.*”* The limitation to movement emerged as a significant event also when loved ones were ill or emotionally distressed and people felt a strong desire to be close to them. Indeed, for two other participants, the worst episode was related to the impossibility to reach and help loved ones who were ill or distressed. *“This friend of mine told me (at the telephone) that her father was in mortal danger (due to coronavirus)*: *in that moment I thought how people are frail (*…*) I did what I could to help her at a distance but I felt impotent*, *useless”* (female, 26, ID#2);

*Impossibility to carry out daily activities during the quarantine*. Three participants recalled the worst episode when they truly realized they could no longer carry out important daily activities during the lockdown (e.g., sports, hobbies). Routines were seen as beneficial for some participants and their interruption had a very negative impact on their psychological well-being. This seems common in people relying heavily on their out-of-home routine, passions, activities, to the point that the “realization” of the quarantine brings along disruptive emotions that tend to last long in their daily experience. *“The first weekend I realized I had nothing to do*, *and for the first time I realized it was not like a week of sick leave or something (*…*) and I felt like in a prison (*…*)*. *Sometimes I still have this sensation of alienation and I don’t know how to make it go away”* (female, 30, ID#18); *“*…*the first day*, *when they said we cannot go out*. *This idea is devastating*, *like having a mental chain (*…*) it was when I read the newspaper and I got panicked*, *I just called all friends and relatives of mine and created pure alarmism*, *it was so bad (*…*) It is still this bad for me*, *I am just crossing days on the calendar (waiting for the end)”* (female, 28, ID#13); note—this participant was the only one in the sample who refused to report a positive event along with the negative one). In other words, the quarantine results in a strong personal constraint that forces people to abandon their daily activities and to rebuild a new daily plan by excluding previous habits. Interestingly, even in the narration of these episodes, people describe the lockdown “like a prison”, emphasizing how the lockdown consisted primarily in a disturbing distortion of activities that normally support emotional stability.

*Alterations to important events*. Six participants were (or will be) able to attend important events but were deeply disappointed by the alterations those underwent because of the quarantine measures (27.27%), ranging from celebrations such as birthdays and bachelor party to the discussion of master degree thesis, which were adapted as video-calls: *“I was waiting for the day and I could see the state of emergency growing in the press (*…*) until the last day I hoped there was another solution but then I had to accept it (*…*) I cannot hide this was not the conclusion I looked forward to (for my education) (*…*) there was not the festive atmosphere I always dreamed of… I had my sister recording it (the ceremony) for Instagram so that my friends could at least see how it went*. *(*…*) For God’s sake*, *I know there could be way more important problems in this situation (*…*) yet there are still anger*, *disappointment*, *bitterness for how it had to end up”* (male, 27, ID#5); The alterations to important events, even if considered inevitable and understandable by the participants due to COVID-19 pandemic, generated negative emotions (e.g., anger) and allow the participants to feel that their *“time and resources were wasted”* (male, 36, ID#17).

#### 2. A sensation of emergency during the quarantine

The second theme relates to the sensation of emergency during the quarantine. Four participants (18.18%) described significant episodes related to the signs of COVID-19 emergency easily identifiable within the life context (*n* = 3) and to the “tilt” or chaos of systems and services due to the pandemic (*n* = 1). These elements generated a “sensation of emergency” and caused negative emotions in participants, such as anxiety, worry, emotional tension. Recognizing quarantine as a clear signal of the national emergency has also led people to consider COVID-19 pandemic not as an event far from personal experience and everyday life, but as a very close danger that significantly impacted their own everyday life.

*The signs of COVID-19 emergency are easily identifiable in the life context*. Events where the signs of the COVID-19 emergency were visible to the participants were classified as the most significant and representative of the quarantine experience, such as the sound of the ambulance in the night, the police cars on the streets with policemen guarding and enforcing the social distancing policies, and the quarrel between people queuing at the post office. For example, a 22 years-old young woman described that one night an ambulance stopped near her home. This immediately triggered the thought that some close person, such as a dear neighbour or relative, may have contracted the virus and was in critical conditions, generating apprehension and fear. Additionally, a 28 years-old woman recalled that, while she was walking her dog, she was stopped by the police and invited to return home. This episode led her to realize the gravity of the situation and made her feel at fault, almost “*like a criminal doing something bad”* (ID#15). These clear signs of the emergency also allowed the interviewee to understand that the COVID-19 situation was serious and allowed to *“realize that it was a really serious thing and we had to stay at home because otherwise (all of this) would be useless"* (female, 28, ID#15). Similarly, one participant talked about the tension easily perceived in ordinary life contexts due to quarantine, such as a supermarket or a post office, which become somewhat alienated by the situation. This participant remembered as the most significant and negative event the day in which he went to the supermarket: *"the row of trolleys at the supermarket parking with people who do not speak*, *harnessed*, *must keep distances*. *There is suspicion in the eyes of people*. *All of them are careful to see if someone would violate the rules [*…*]*. *I see that even the most normal things are different from the usual"* (male, 30, ID#20). Among people, escalation of tension may occur, as in the episode narrated by the participant who witnessed an altercation between two other customers, which he felt as the result of stress: *"I think it is the demonstration of the difficult moment that you are experiencing*. *it takes little to light up compared to normal situations in which maybe the reaction would have been different"* (ID#20). This led the participant to feel *"annoyance and irritation [*…*] (those people) not respecting the rules in a situation where they are trying to protect us*… *it annoyed me"* (ID#20).

*The “tilt” or chaos of systems and services due to the pandemic*. An episode remembered by one participant was centered on the "tilt" and chaos that the quarantine brought along and which conveyed a “sensation” of emergency. This theme clearly emerged in the significant episode told by a 29 years-old woman about transport services that were suspended and provided uncertain or inaccurate information. This complicated the return of her brother to his home, generating anxiety and concern about what could have happened. *"My brother had to move to reach his own family and did not understand how to do it*, *how to move*. *It was the beginning of the quarantine*, *so it was not clear if he could move and how he could do it*. *I remember this great anxiety in which it was not possible to figure out what to do*… *my attempt to help him by looking at flights*, *trains*, *which were immediately canceled*… *this enormous confusion linked to this travel he had to do*… *it was necessary but no one knew how to help him"* (female, 29, ID#6).

#### 3. Relationships during the quarantine

The third theme related to the management of relationships during the quarantine. Quarantine led people to "test" their relationships, forcing people to live together in the same space for a long time, or on the contrary being forcibly distanced. At the same time, common relationship issues were sometimes exaggerated during the quarantine (*n* = 6; 27.27%).

*Putting relationships to the test*. Living with the partner, although it may appear positive for some people with a satisfactory relationship, is a source of frustration, anger and nervousness for others, to the point that in some cases the relationship literally ended. This is the case of a young woman who described the detriment of her love relationship as the most negative episode of her quarantine: *“living together in this particular moment has brought to light unresolved issues in our relationship that we have been carrying out for some time”* (female, 32, ID # 1). The impossibility of having personal spaces and having to look after another person with whom the relationship had become problematic was extremely difficult during the quarantine. Additionally, it is possible that people experienced “stress and panic”, resulting in family misunderstandings.

This was emphasized by the limited spaces in which some people were forced to live during the pandemic. This can lead people to constantly look for an external outlet (e.g., in the garden or on the balcony) to "*breathe some fresh air*" (male, 37, ID#16) and take personal space. For example, one participant reported frequent arguments with her parents and the difficulties in sharing personal spaces with them and the effort to find ways to isolate herself *“in the backyard*, *or in some places where nobody could come*, *to just be completely alone”* (female, 21, ID#3).

*Preoccupation for others*. The quarantine has led people to worry about the health of their loved ones and anxious that they may contract the virus. For example, a young woman in the sample reported when she was informed by her mother that COVID-19 “arrived” in the long-term care nursing homes where the mother worked: *“I was so scared*, *I feared that something could happen to her and started having catastrophic thoughts (*…*) had some nights I couldn’t sleep*, *thinking something could happen to her”* (female, 34, ID#12). Similarly, another participant received a call about the elderly grandmother and aunt in the nursing home being in critical conditions, and experienced preoccupation, impotency, and sadness for the “*thought of their solitude*” (female, 31, ID#22).

Only one participant in the sample reported a job-related episode, but it could be reported into this category because the negativity was related to preoccupation for the loved one’s job, that was put at risk by the lockdown: “*My husband is on forced vacation because its company is no longer receiving commissions from clients (*…*) I am worried as well because of seeing him so sad*, *especially about the uncertain future now that our daughter is about to come into the world (*…*) I am just trying to be positive and to not show that I am worried like him*” (female, 31, ID#9).

### Positive events

Participants included in this study were also invited to describe a significant positive episode related to the lockdown. It is interesting to note that one young woman (29-years old) was unable to remember a positive significant event. Thus, a total of 21 critical positive incidents were collected and coded. Four themes and seven sub-themes emerged from the positive episodes.

#### 1. Quarantine as an opportunity to take care of personal relationships

The first theme relates to the quarantine as an opportunity to take care of personal relationships. Twelve of the 21 participants (57.14%) described significant episodes related to the improvement of relationships with the partner (*n* = 1), family (*n* = 7), or friends (*n* = 4). In this sense, quarantine was seen as an opportunity to spend more time with the loved ones, experiencing also new ways of communication (e.g., new technologies). This allowed people who live in different cities to feel "closer, even if very far away".

*Improvement of relationships inside home*. Six participants recalled positive episodes related to the renewed opportunity to stay in touch with their loved ones. A young woman (31-years old) recalled as the most positive event the opportunity to spend a lot of time with her partner. Often, due to the busy working life, the relationship can be overlooked. Instead during the lockdown, the woman lives the whole day with her loved one, experiencing positive emotions and feeling supportive during the unpleasant experience of the pandemic. The couple discovered new activities and shared interests: *“we bought a puzzle (never did in our life)*… *with great emotional frustration we gave ourselves to the construction of this immense puzzle*… *we have filled our days with many significant episodes of activities done together in which the important thing was not so much the content of the activity*, *but to fill our time constructively and cooperatively*.*”* (female, ID#7).

The relationship with the loved ones was strengthened not only by finding new time and spaces within the quarantine, but also by the shared experience of being part of the same strange situation, which probably enhanced reciprocal empathy and sense of belongingness:*“Usually I am the type of guy who participates very little in conversation with my family*, *for example at the dinner table I just respond to questions by my parents and my aunt (*…*) these days my participation increased a lot (*…*) Like*, *we should enjoy what we have in full*, *making less plans for the future*, *enjoying the moment and the day-to-day life”* (male, 27, ID#5). In other words, the quarantine results not only in being “constricted together” inside the home, but possibly as a catalyst for renewing social interaction, sharing and co-elaboration of feelings related to the situation. When one’s own and others’ safety is not put at risk directly (i.e., no one in the family unit is infected), new space for developing significant relationships can be found within the quarantine experience.

*Use technologies to stay in touch with distant loved ones*. When not living together, participants often referred to the usage of digital communication technologies (e.g., video call services) as a fundamental tool to cultivate significant relationships: “*I have a big family with many cousins*, *I have eleven cousins and each of them has a partner and children*… *so a big family*! *One of these cousins has turned fifty and we have been able to connect all to this video call service in some way to celebrate his birthday*. *It was a surprise because this cousin’s sister put the computer inside a box and when he opened it*, *such as it was a gift*, *he saw all these squares (the video windows) with all of us cousins connected*. *This made us feel very close despite the distance*… *(*…*) and like*, *everyone was in the same situation*. *Knowing that it’s not just me who is like this but everyone is like that*.*”* (female, 32, ID#1). Besides cultivating already established relationships, people find themselves in the necessity to develop communication resources. This may lead them to renovate or rediscover contact with acquaintances the relationship with was occasional or almost nonexistent before: “*I mean*, *you connect with people that you never used to hear from”* (male, 27, ID#21). Maybe paradoxically, the experience of the quarantine becomes the occasion to develop social relations, in virtue of the fact that everybody is facing the same challenges as well as similar emotions.

Other participants report similar episodes, characterized by video-calls with friends that allow to celebrate occasions (e.g., one’s birthday) or to recover meeting habits thanks to the mediation of communication technologies (ID#8, ID#19, ID#20).

#### 2. The working career will continue despite the pandemic

The second theme refers to the working area. In a time of job uncertainty in which many enterprises are forced to close, job security is very good news. During the lockdown, three participants (14.29%) described significant events related to the discovery of new working activities (*n* = 1) or the confirmation of their employment contract (*n* = 2).

*Discovery of new working activities during the quarantine*. Quarantine allowed a participant to get involved in a new working activity. Given the great psychological impact related to the COVID-19 pandemic, a young psychologist (33-years old) started to collaborate as an emergency psychologist. This activity allowed the woman to perceive a sense of well-being and usefulness in such a difficult moment, but also a confirmation of personal abilities.

*Confirmation of the employment contract*. Two subjects described the confirmation of the employment contract as the most positive experience lived in the quarantine. A young woman described the phone call in which she received confirmation of her employment contract as the most positive episode: *“This has been a great personal success for me (*…*) because I have been on my job route just from a year and I am still insecure*, *I had no certainties”* (female, 26, ID # 2).

The quarantine led many people to experience a strong sense of concern and uncertainty for the future. The confirmation of the employment contract allowed people to regain serenity and a great motivation at work. While confirmation of one’s job is certainly a positive news during normal times too, it is felt as even more empowering in a period filled with fear and uncertainty: people may understand they are valuable, useful, skillful, worth to receive confirmation in spite of a context in which nothing could be taken for granted. In other words, the quarantine could suddenly become an obstacle that has been already overcome in part, at least at a personal level, in that professional achievement in it is even more valuable and reassuring.

#### 3. A new value for everyday activities

The third theme refers to the value that people attributed to simple life activities, whose pleasure was rediscovered during the lockdown. Five participants described the quarantine as an opportunity to give a new value and meaning to everyday gestures (*n* = 1) or to discover new activities and hobbies (*n* = 4).

*A sense of freedom*. After 36 days of quarantine, one young man recalled as the most significant and positive episode of the lockdown the day in which he had the possibility to just drive his car to go to the supermarket. It seems that the quarantine has allowed people to give a different meaning to simple daily activities and new value to everyday gestures: “*Just taking the car*, *such as all those gestures you take for granted or even annoyed you (I never liked going to buy groceries)*… *that day*, *that gave me a sense of freedom*. *At first it was weird*, *I was like “*…*do I still know how to drive*? *Am I still able to*?*”*…*weird at first*, *but then pleasant*. *A little glimpse of normality for me*. *Returning to normality for ten minutes*. *(*…*) it was like diving back into normality after it was run over*.*”* (male, 36, ID#17).

*Modifying or discovering activities*. Other activities were modified or discovered during the quarantine, ranging from physical exercise to trying new hobbies. Four participants explicitly reported the quarantine as a positive experience, with room for “sense of gratification”, “relax”, “creativity and cooperation”. For example, one participant recovered a tradition with her husband while staying at home: *“The first saturday*, *because usually on saturday we use to have dinner out (*…*) I just laid the table with candles*, *get dressed like if we had to go out*, *and we had dinner and it was nice (*…*) now we created this new routine we do any saturday and sunday (*…*) usually I love to go out for dinner and I thought it would have been depressing to do that at home*. *Instead*, *I liked it very much and I was surprised I could find my happiness in little things different from usual”* (female, 28, ID#15).

While the “happiness from little things” concept may appear trivial, it emerged in participants’ accounts. Staying at home for longer periods of time and reducing stimuli and commitments from outside (coupled with a vague sense of danger but no immediate threats to safety), gave people more space to enjoy everyday gestures and activities, sometimes finding beauty and satisfaction in them.

#### 4. Quarantine as an opportunity to take a break from the normal routine

Another theme that emerges from this study is the quarantine as an opportunity to take a break from the normal routine.

*Take your time*! One participant described the quarantine as a peaceful moment in which he had the possibility to “detach his mind” from the normal routine and “take time for himself”. Again, this positive consideration was related thanks to the shared experience aspect of the lockdown: *“I am aware that I am not the only one who does not leave the house*, *but this is a shared situation*. *Knowing that everyone lives this situation*, *allows me to live in a relaxed way”* (male, 30, ID#14).

## Discussion

To our knowledge, this is the first study to report qualitative descriptions of significant events by citizens during the COVID-19 quarantine. 22 participants were interviewed about specific positive and negative events (“incidents”) deemed representative of their lived experience of the quarantine, in order to provide information on their experience as a whole. Recent research and reviews explored the effects of quarantine on mental health (e.g., its effects on anxiety and other psychopathological conditions). However, there is still a lack of specific information on the impact of COVID-19 containment measures on the everyday life of citizens, as well as on their perception of the current situation and the future.

### Main outcomes

In this study, we gathered experiences from Italian people who experienced COVID-19 lockdown. Significant positive and negative events were collected through audio-recorded interviews and participants reported 22 negative and 21 positive critical events related to the lockdown. These events were grouped into three main categories for negative events and four main categories for positive events.

Social relationships were particularly affected by the quarantine and emerged in a huge part of the events’ sample. On the negative side, family and love relationships that already had relational or communicational issues may become unsustainable during the forced, continuous cohabitation, sometimes even leading to relationship end. On the positive side, social interactions that were limited during pre-lockdown life emerged as enriched by the amount of time spent together. Moreover, digital communication technologies emerged as an invaluable tool to cultivate distant relationships and recover social activities. This allowed people who live in different cities to feel "closer, even if very far away".

Secondarily, the quarantine affected everyday life in terms of activities and commitments. Negative events in this category regarded the inability to reach a specific important event, to help loved ones in need, to carry out daily activities due to the lockdown. The recollection of these episodes was associated with emotions of anger and disappointment. Participants referred to the quarantine as “prison” multiple times, emphasizing how the lockdown consisted primarily in a limitation to freedom and a disturbing distortion of those activities that normally support emotional stability. Yet, on the positive side, staying at home allowed many participants to get creative in order to maintain family habits and traditions, with the consequence of obtaining unexpectedly positive consequences. Additionally, it seems that quarantine has allowed people to attribute a new meaning to simple daily activities and to everyday gestures. Participants remembered episodes in which they rediscovered activities with a renewed, positive feeling due to successful adaptation. These activities covered a wide range, from physical exercise to trying new hobbies or to take a break from the normal routine, but also to “detaching the mind” and “finally taking some time for myself”.

Unexpectedly, the work sphere has emerged generally little in participants’ accounts. Receiving the confirmation of job security or discovering new working opportunities was a positive episode for some participants, so that the conditions for future stability were created exactly in virtue of the emergency situation. Conversely, no negative events specifically related to work were reported in the sample, besides one participant who expressed concern about a loved one’s unstable job situation. One possible explanation is that, while many jobs and personal finances are put at risk in the COVID-19 pandemic, many are still waiting to receive news about their professional situation. Or alternatively, it is possible that some individuals are stressed or worried about their work and financial situation, but this stress is still not tied to a specific event (e.g., being fired) that could be reported as the most negative/displeasant.

A particularly interesting theme that emerged in this study is the “sensation of emergency”. Participants remembered episodes in which the signs of emergency are easily identifiable in the life context. This is something inherently different from being annoyed by alterations in habitual activities: it relates to the sudden recognition of the emergency on a personal level, accompanied by a strong emotional activation, typically in terms of fear or anxiety, or sadness because of realizing the fragility of oneself and of the dear ones. The sensation of emergency is related to environmental signs that burst into the everyday routine, ranging from the close siren of the ambulance in the night to being notified of a loved one being in distress or danger. Recognizing quarantine as a national emergency on the basis of signs in everyday life has also led people to consider COVID-19 not as an event "distant" from personal experience, but as a very close danger that significantly impacted their own life. The sensation of emergency appears also connected often to the feeling of impotence. This way, it appears that the emergency situation do not only affect those who are directly involved by the health concerns (e.g., by contracting the virus), but also those who experience the situation from the safety of their homes, when external stimuli remind them of the concreteness of the risks and of their own limited agency over the situation.

From a psychological point of view, the signs of emergency could lead people to experience anxiety-provoking hyperarousal symptoms related to negative sensory or emotional issues that can persist even after the end of the quarantine. In complex situations, the intrusive thoughts of the signs of emergency may represent a traumatic experience for people, leading to fear and avoidance behavior [9, 33, 34].

Finally, the research has shown that participants experienced positive episodes as well during the quarantine, mostly related to having the occasion to cultivate intimate relationships or to discover new activities and hobbies, or even just to enjoy some time for oneself. The analysis of positive events could be interesting as well in terms of mental health. Although no participants in the sample said to be “happy” about the quarantine, it clearly emerged that the unique situation had some positive sides, with an emotional charge equal to that of their negative counterparts. Some journalistic and popular sources are reporting on the “cabin syndrome” phenomenon (not to be confused with the opposite “cabin fever” or the stress related to forced confinement) [35, 36]: it is possible that some people got attached to the sense of security conveyed by their own homes, as well as to the positive aspects of this experience. While scientific data are still lacking on this phenomenon, it is possible that returning to normal, social life will be more emotionally costly for some individuals, leading to an emergent mental health issues.

### Implications

The COVID-19 quarantine represents a unique phenomenon and its consequences on people’s lives, on public mental health, and on social relationships are still to be understood. The lockdown led to the alteration of many everyday activities, ranging from education to market behavior, and interrupted travel around the world. This led many people to lose their certainties, interrupting their routine and forcing them to build new habits. In addition to these, many people have experienced (1) more or less traumatic or disturbing experiences, (2) events that may have an effect on their economic or family-relationship stability, (3) complex events and emotions without an adequate social support, and (4) changes in their routines and habits that led them to reflect on their life priorities.

In general, this study helps to identify which life areas have been affected the most by the isolation and quarantine, so to orient future psychological support and mental health policies. Also, in terms of positive events, it contributes to the understanding of the positive resources people have been able to employ in order to manage stressful factors, so to inform a societal comprehension of the lockdown phenomenon from the personal viewpoint of citizens.

This research adds a new dimension to our understanding by conveying a thematic account of the psychological experience of Italians who were experiencing the quarantine. Data can be used to prefigure psychological support in the long-term aftermath of the COVID-19. For example, some family and couple relationships may emerge injured or weakened by the quarantine experience, because the forced cohabitation or the prolonged distance have worsened pre-existing relationship issues which can lead to conflict and misunderstanding. In terms of mental health support, mental health professionals may take into consideration the importance of discerning specific issues that emerged from the quarantine and the pandemic experience, in order to develop treatment resources grounded in the uniqueness of the post-pandemic scenario.

Another area of development is that of mobile applications and internet-based resources that provide users with structured tools to keep trace of their own physical and mental health [37, 38]: while tracking apps are currently used to control the spreading of the virus, there is still little to none tools focused on the experience of the quarantine along with its current and future risks for mental health. The identification of individuals’ issues could give health applications developers new indications in terms of life areas to support by eHealth solutions.

In terms of the “sensation of emergency”, future research may explore whether such emotional events could reach the status of trauma, with psychopathological consequences in the long-term period. As in other forms of pandemic (e.g., SARS in Canada) in which people experienced symptoms of trauma similar to that of natural disaster or terrorist attack [39], the COVID-19 pandemic can expose people to traumatic experiences. Unexpected emergencies and traumatic circumstances during the quarantine (e.g., the death of loved ones; the forced social distancing) may be related to intrusive fantasies and thoughts connected to the trauma, dissociations, and emotional burden. This could have a negative impact on social, working and relational functioning [40]. It is important to take into account individual differences (e.g., personality traits) [41, 42] and the possible traumatic consequences of the pandemic and rethink mental health provisions and psychological support for people in order to promote a reconnection between dissociated parts and the elaboration of traumatic experiences [43–46].

### Strengths and limitations

The strengths of this study include a focus on the positive and negative experiences during the COVID-19 quarantine. As in other studies [e.g., 21, 47], data were obtained by restricting participant accounts to only events in which they were directly involved during the lockdown through the Critical Incident Technique. Rather than collecting general opinions on the quarantine, this technique focuses on relevant *episodes* representing individuals’ experience, without forcing them into predetermined categories. Another strength is that we carried out this research during the quarantine itself, collecting information on individuals’ life within the exact context and period of interest. This allows us to collect feelings and memories at the time of the event, minimizing the risk of distortions and forgetfulness with direct access to the recent memory.

The primary limitation of the research is the sample size along with the qualitative research method. The sample was determined on the basis of data saturation but, although informative, the data of this study cannot be generalized to the whole national or international population. Participants are well balanced for gender, but not for age and participants were also mostly based in northern Italy, which has been particularly affected by COVID-19 compared with the central and the south, and therefore data may be not representative of the experiences at a national or international level. In addition, interviews conducted via Skype present additional barriers and relevant information may be missed. Some respondents found it easier to describe the negative episode experienced in the quarantine rather than the positive event. Indeed, one young woman could not be able to describe a positive event related to the lockdown.

Finally, by employing a method focused on specific episodes (“incidents”), the research was able to identify meaningful and personal examples of life within the quarantine, instead of general abstract opinions. This considered, it is possible that important components of individuals’ experience were not identifiable by the methodological approach, for example because they were not tied to specific events (e.g., overall mood).

## Conclusion

This study aims to investigate the significant negative and positive events that people experienced during the COVID-19 quarantine in Italy. 22 Italians who did not contract COVID-19 were invited to participate in semi-structured interviews employing the Critical Incident Technique. Data can be used to prefigure psychological support in the long-term aftermath of the pandemic and to orient future research on personal experiences.

Future research could employ analogous event recollection methods but focus on specific populations, for example those who contracted the virus, those who had to experience quarantine as fragile subjects (e.g., chronic patients) or on other national contests, in order to extend the information on the quarantine experience and its possible long-lasting effects.

## Supporting information

S1 Appendix Interview guide. (DOCX)

S2 Appendix Checklist for fulfillment of consolidated criteria for reporting qualitative studies (COREQ). (DOCX)
