# Supplementary material for: In the eye of a quiet storm: A critical incident study on the quarantine experience during the coronavirus pandemic
Source: PLoS One. 2021 Feb 17;16(2):e0247121. doi: 10.1371/journal.pone.0247121 (PMC7888600; doi:10.1371/journal.pone.0247121)
Supplement: S1 Appendix — (DOCX) [file pone.0247121.s001.docx]

**INTERVIEW GUIDE (Italian)**

Vorrei farle alcune domande sulle sue esperienze più significative vissute durante questo periodo di quarantena causa COVID-19. Le chiedo di fornirmi alcune informazioni specifiche su episodi che si sono verificati in questo periodo.

Nello specifico, tra le esperienze che hai vissuto durante questa quarantena, qual è l’**episodio più negativo/episodio più positivo**?

**Prompts:**

*Descriva la circostanza e la natura di questo evento*

*Mi aiuti a capire come l’esperienza ha avuto luogo, cosa ha fatto, cosa è successo dopo, quante volte si è verificato l’episodio e quali figure erano coinvolte*

*Mi aiuti a capire perché considera questo episodio come rilevante. Cosa pensa di questo episodio? Cosa ha pensato quando ha vissuto questo episodio? Quali sono gli elementi che lo hanno reso negativo/positivo?*

*Emozioni provate- Cosa hai provato durante questo episodio?*

**INTERVIEW GUIDE (English)**

I would like to ask you some questions about the most significant experiences during this quarantine caused by COVID-19 spread. I am asking you to provide me with specific information on episodes that happened during this period.

Specifically, among the experiences that you lived during the quarantine, what is the **most negative episode/the most positive episode**?

**Prompts:**

*Describe the context and nature of this event*

*Help me understand how the event took place - what did you do, what happened before/later, how many times did this happen (more than one?) which other persons were involved?*

*Help me understand why this episode is relevant to you - What do you think about it? What did you think at the time? What aspects made the event positive/negative?*

*Emotions - what did you feel during/after this episode?*
