# Supplementary material for: In the eye of a quiet storm: A critical incident study on the quarantine experience during the coronavirus pandemic
Source: PLoS One. 2021 Feb 17;16(2):e0247121. doi: 10.1371/journal.pone.0247121 (PMC7888600; doi:10.1371/journal.pone.0247121)
Supplement: S2 Appendix — (DOCX) [file pone.0247121.s002.docx]

**Durosini, Triberti, Savioni, & Pravettoni – In the Eye of a Quiet Storm: A Critical Incident Study on the Quarantine Experience During the Coronavirus Pandemic – COREQ Checklist**

| **No Item** | **Guide questions/description** | **Response** | **page no.** |
| --- | --- | --- | --- |
| Domain 1: Research team and reflexivity | | | |
| *Personal Characteristics* | | | |
| 1. Interviewer | Which author/s conducted the interview or focus group? | ID, ST, LS | 6 |
| 1. credentials | What were the researcher’s credentials? E.g. PhD, MD | ID, ST: PhD  LS: PhD student | - |
| 1. occupation | What was their occupation at the time of the study? | ID, ST: post doc researchers  LS: PhD student | - |
| 1. gender | Was the researcher male or female? | ID, LS = female  ST = male | 6 |
| 1. experience and training | What experience or training did the researcher have? | All the researchers had previous experience in interviewing and qualitative data analysis and received academic training in qualitative research methods;  ID had used the Critical Incident Technique multiple times since her PhD research | 6 |
| 1. relationship established | Was a relationship established prior to study commencement? | No; the researchers did not know the participants they interviewed. The researchers made sure to create a welcoming and safe interview environment through brief introductions to each individual interview | - |
| 1. participant knowledge of the interviewer | What did the participants know about the researcher? e.g. personal goals, reasons for doing the research | The participants were informed of the main aim of the research only, i.e., to analyze citizens’ experience of the quarantine by selection of notable episodes. | 6 |
| 1. Interviewer characteristics | What characteristics were reported about the interviewer/facilitator? e.g. Bias, assumptions, reasons and interests in the research topic | The researchers were quarantined themselves; when designing the research, they made sure to adopt an objective and unbiased approach to the interviews, in order to not influence the participants in their response with their subjective experience of the quarantine. Besides that, the research had an exploratory and open approach, and all the research declared the absence of specific expectations or interests | 6-7 |
| Domain 2: study design | | | |
| *Theoretical framework* | | | |
| 1. methodological orientation and theory | What methodological orientation was stated to underpin the study? e.g. grounded theory, discourse analysis, ethnography, phenomenology, content analysis | Critical Incident Technique | 6-7 |
| 1. sampling | How were participants selected? e.g. purposive, convenience, consecutive, snowball | Purposive sample | 5 |
| 1. method o approach | How were participants approached? e.g. face-to-face, telephone, mail, email | online interview due to the COVID-19 pandemic; the interviews have been performed via Skype | 6-7 |
| 1. sample size | How many participants were in the study? | 22 participants | 5 |
| 1. non-participation | How many people refused to participate or dropped out? Reasons? | 5 participants refused to participate in the study. The reasons are shyness and not time available | 5 |
| 1. setting of data collection | Where was the data collected? e.g. home, clinic, workplace | both researchers and participants were in their homes due to the ongoing COVID-19 quarantine; the interviews have been performed via Skype | 5 |
| 1. presence of non-participants | Was anyone else present besides the participants and researchers? | Participants were asked to isolate themselves as possible during the online interviews; researchers as well managed to conduct the interviews when isolated from cohabitants (when present) | 5 |
| 1. description of sample | What are the important characteristics of the sample? e.g. demographic data, date | age 20-40, italian citizens, quarantined for COVID-19, not infected by corona virus | 5 |
| 1. interview guide | Were questions, prompts, guides provided by the authors? Was it pilot tested? | yes; the research was not pilot tested | Supporting Information |
| 1. repeat interviews | Were repeat interviews carried out? If yes, how many? | no | - |
| 1. audio/visual recording | Did the research use audio or visual recording to collect the data? | audio recording | 5 |
| 1. field notes | Were field notes made during and/or after the interview or focus group? | no | - |
| 1. duration | What was the duration of the interviews or focus group? | each interview lasted around 20-40 minutes | 7 |
| 1. data saturation | Was data saturation discussed? | Yes | 20 |
| 1. transcripts returned | Were transcripts returned to participants for comment and/or correction? | No | - |
| Domain 3: analysis and findings | | | |
| *Data analysis* | | | |
| 24. Number of data coders | How many data coders coded the data? | two, ID and ST | 7 |
| 25. Description of the coding tree | Did authors provide a description of the coding tree? | Results section describes categories and subcategories of the results | 8-16 |
| 26. Derivation of themes | Were themes identified in advance or derived from the data? | Themes were derived from the data | 8-9 |
| 27. Software | What software, if applicable, was used to manage the data? | We did not use a software for data analysis | - |
| 28. Participant checking | Did participants provide feedback on the findings? | No | - |
| *Reporting* | | | |
| 29. Quotations presented | Were participant quotations presented to illustrate the themes / findings? Was each quotation identified? e.g. participant number | Yes and yes | 9-16 |
| 30. Data and findings consistent | Was there consistency between the data presented and the findings? | Yes | 16-18 |
| 32. Clarity of major themes | Were major themes clearly presented in the findings? | Yes | 8-16 |
| 31. Clarity of minor themes | Is there a description of diverse cases or discussion of minor themes? | Yes | 9-16 |
